# Prediction of breast cancer Invasive Disease Events using transfer learning on clinical data as image-form

**DOI:** 10.1371/journal.pone.0312036

**Published:** 2024-11-21

**Authors:** Annarita Fanizzi, Samantha Bove, Maria Colomba Comes, Erika Francesca Di Benedetto, Agnese Latorre, Francesco Giotta, Annalisa Nardone, Alessandro Rizzo, Clara Soranno, Alfredo Zito, Raffaella Massafra

**Affiliations:** I.R.C.C.S. Istituto Tumori “Giovanni Paolo II”, Bari, Italy; Local Health Authority Caserta: Azienda Sanitaria Locale Caserta, ITALY

## Abstract

**Background and objective:**

Detecting patients at high risk of occurrence of an Invasive Disease Event after a first diagnosis of breast cancer, such as recurrence, distant metastasis, contralateral tumor and second tumor, could support clinical decision-making processes in the treatment of this malignancy. Though several machine learning models analyzing both clinical and histopathological information have been developed in literature to address this task, these approaches turned out to be unsuitable for describing this problem.

**Methods:**

In this study, we designed a novel artificial intelligence-based approach which converts clinical information into an image-form to be analyzed through Convolutional Neural Networks. Specifically, we predicted the occurrence of an Invasive Disease Event at both 5-year and 10-year follow-ups of 696 female patients with a first invasive breast cancer diagnosis enrolled at IRCCS “Giovanni Paolo II” in Bari, Italy. After transforming each patient, represented by a vector of clinical information, to an image form, we extracted low-level quantitative imaging features by means of a pre-trained Convolutional Neural Network, namely, AlexNET. Then, we classified breast cancer patients in the two classes, namely, Invasive Disease Event and non-Invasive Disease Event, via a Support Vector Machine classifier trained on a subset of significative features previously identified.

**Results:**

Both 5-year and 10-year models resulted particularly accurate in predicting breast cancer recurrence event, achieving an AUC value of 92.07% and 92.84%, an accuracy of 88.71% and 88.82%, a sensitivity of 86.83% and 88.06%, a specificity of 89.55% and 89.3%, a precision of 71.93% and 84.82%, respectively.

**Conclusions:**

This is the first study proposing an approach which converts clinical information into an image-form to develop a decision support system for identifying patients at high risk of occurrence of an Invasive Disease Event, and then defining personalized oncological therapeutic treatments for breast cancer patients.

## Introduction

Although breast cancer death rates have steadily decreased in recent years, breast cancer is still a leading cause of cancer death in women worldwide [1]. Recently, significant progress has been made to improve the therapeutic outcome with the introduction of novel therapies [2]; however, the possibility of accurately predicting the occurrence of an Invasive Disease Event (IDE) after a first diagnosis of breast cancer, such as recurrence, distant metastasis, contralateral tumor and second tumor, remains an unmet clinical need.

So far, in the state-of-the-art, several studies have demonstrated the central role of breast cancer subtypes on both cancer prognosis and treatment effectiveness [3–6]. According to their molecular subtype, patients are assigned to a specific adjuvant treatment after surgery, thus saving low-risk patients from unnecessary and/or potentially toxic pharmacological treatments. Besides, several prognostic online tools, such as Predict and Adjuvant, are currently available for estimating the survival rate of patients depending on the selected therapeutic option. Though these tools are usually adopted in clinical practice, different validation studies have demonstrated their accuracy is not reliable on all subsets of patients [7–9].

Within this scenario, predictive models able to achieve a well-balanced compromise between reliable therapy response predictions and cost-effectiveness are urgent. Accurate tools based on both molecular and protein markers have been already proposed, but they are expensive and not all centers are equipped with laboratories capable of performing this analysis [10]. On the other hand, the great impact that artificial intelligence has had in the biomedical field [11–13] has encouraged the design and development of machine learning models to support clinical decision-making processes in the treatment of breast cancer [14–18]. These automated models are trained on both clinical and histological data commonly collected in clinical practice, and do not require additional expansive information. However, even though several studies have proposed models achieving encouraging results, these are not yet suitable in clinical practice due to either not great performances or the absence of a proper validation on consistent datasets [19–25]. Hence, the prediction of an invasive disease event after a first breast cancer diagnosis is still a challenging task [26–29].

Recently, we proposed a novel ensemble machine learning classification model able to predict the occurrence of IDEs after a primary breast tumor, such as recurrence, metastasis, contralateral and second tumors at both 5- and 10-years follow-ups [30]. IDE prediction, despite being less investigated in literature, is of great interest in the adjuvant clinical trial setting due to treatment-related causes may play a role in the occurrence of second tumors or contralateral breast cancers [31, 32]. Though the proposed approaches [30, 33] were the result of a complex classification technique based on the concept of voting among multiple models and was validated on a substantial sample, performances were not acceptable for the application in clinical practice. Performances achieved by these models are affected by the presence of the so-called confounding patients having the same clinical and histological characteristics as other patients but responding differently to the adjuvant treatment [33].

According to other models proposed in literature [25], even the most cutting-edge machine learning algorithms turned out to be unsuitable for modelling the complexity of this problem.

With the aim of overcoming these limits, in this study we propose a model which relies on pre-trained Convolutional Neural Networks (CNNs) to analyze images obtained transforming each patient, represented by a vector of clinical information, to an image form. Particularly, the image transformation procedure reproduces an approach employed until now to analyze only genomic data [34], thus this is the first time this procedure is applied on clinical and histological characteristics and with the purpose of predicting an IDE after a breast cancer diagnosis. Actually, to the best of our knowledge, this is the first study which propose an approach converting clinical information into an image-form, allowing to exploit pre-trained CNNs capabilities for extracting latent information enclosed in the structured data. Pre-trained CNNs refer to a transfer learning approach which lets to extract quantitative imaging features from images according to which the networks have previously learned during training on a very huge (millions) number of images of different nature [35, 36]. Thus, the knowledge acquired from the network during this training phase has been then transferred and applied on images associated to our patients, with the purpose of predicting the risk of occurrence of an IDE after a first breast cancer diagnosis within both 5-year and 10-year after diagnosis.

## Material and methods

### Experimental dataset

This retrospective study was approved by the Ethics Committee of the Istituto Tumori “Giovanni Paolo II” of Bari, Italy, and involves 696 female patients registered for a first invasive breast cancer diagnosis in the period 1992–2021. A written ‘informed consent’ for publication was collected for all the patients involved in the study, except for patients who are dead or not reachable, as it is a retrospective study, and the data were accessed for research purposes on February 7^th^, 2023. Afterwards, a data de-identification process was performed in order to not allow to identify individual participants during or after data collection. Patients who underwent neoadjuvant chemotherapy and/or had carcinoma in situ and/or had metastasis *ab initio* were not included in this study. Besides, only patients who had an IDE within 5 years from the first tumor diagnosis or patients who had not an IDE after the first tumor and with at least 5-years of follow-up were involved in this study, for a total of 168 recurrence IDE cases and 528 control cases (non-IDE cases). Then, with the aim of also predicting the possibility for a patient to have a relapse within 10 years from the first breast cancer diagnosis, we defined a sub-sample of the experimental dataset which counts 626 patients having either an IDE within 10 years from the first tumor diagnosis or having not an IDE and with a follow-up of at least 10 years, for a total of 251 IDE cases and 375 control cases. The difference between the two sample sizes is due to the absence of a 10-years follow-up for some patients belonging to the first dataset. Breast cancer clinical and histopathological data, as well as chemotherapy information, were collected from the patients’ medical records. For each patient, a total of 28 features were compiled, comprising age at diagnosis, previous tumors (values: absent/present), breast site of the tumor (abbr. breast, values: right/left), histological type (values: ductal, lobular, others), intraductal component (values: absent, not typed, G1, G2, G3), multifocality (values: absent/present), angioinvasion (values: absent, focal, extensive, not typed), estrogen receptor expression (abbr. ER, % value), progesterone receptor expression (abbr. PgR, % value), cellular marker for proliferation (abbr. ki67, % value), human epidermal growth factor receptor-2 score (abbr. HER2/neu, values: 0, 1, 2, 3), human epidermal growth factor receptor-2 (abbr. HER2, values: negative/ positive), histological grade (abbr. grading, values: G1, G2, G3), tumor size (abbr. diameter, values: T1a, T1b, T1c, T2, T3, T4), lymph-node status (values: N0, N1, N2, N3), having performed the sentinel lymph-node biopsy (abbr. SLNB, values: not performed, negative, positive), having performed the axillary lymph-node dissection (abbr. ALND, values: not performed/performed), eradicated lymph-nodes, metastatic lymph-nodes, type of surgery (abbr. surgery, values: mastectomy/conserving-surgery), having received chemotherapy (abbr. chemotherapy, values: no/yes), chemotherapy scheme (values: not received, C1—anthracycline and taxanes, C2—anthracycline, C3—taxanes, C4—CMF, C5—other), having completed chemotherapy treatment (abbr. chemotherapy completion, values: yes/no), having received trastuzumab (abbr. trastuzumab, values: no/yes), having completed trastuzumab treatment (abbr. trastuzumab completion, values: yes/no), having performed hormone therapy (abbr. hormone therapy, values: no/yes), hormone therapy scheme (values: not received, H1—tamoxifen, H2—Lh-Rh, H3—tamoxifen and Lh-Rh, H4—Aromatase Inhibitor, H5—tamoxifen and Aromatase Inhibitor, H6—Lh-Rh and Aromatase Inhibitor, H7—other) and having completed hormone treatment (abbr. hormone therapy completion, values: yes/no).

An overview about the sample clinical properties is provided by Table 1.

**Table 1:** Clinical features distribution over the study population.

Feature	Distribution	Feature	Distribution
**Overall**	696; 100%	N1 (abs; %)	243; 34.9%
**Age at diagnosis**		N2 (abs; %)	53; 7.6%
Median; [q_1_, q_3_]	52; [45, 62]	N3 (abs; %)	32; 4.6%
**Previous tumors**		NA (abs; %)	7; 1.0%
Absent (abs; %)	678; 97.4%	**SLNB**	
Present (abs; %)	18; 2.6%	Not performed (abs; %)	537; 77.1%
**Breast**		Negative (abs; %)	98; 14.1%
Right (abs; %)	348; 50.0%	Positive (abs; %)	48; 6.9%
Left (abs; %)	346; 49.7%	NA (abs; %)	13; 1.9%
NA (abs; %)	2; 0.3%	**ALND**	
**Histological type**		Not performed (abs; %)	95; 13.7%
Ductal (abs; %)	587; 84.4%	Performed (abs; %)	587; 84.3%
Lobular (abs; %)	56; 8.0%	NA (abs; %)	14; 2.0%
Others (abs; %)	53; 7.6%	**Eradicated lymph-nodes**	
**Intraductal component**		Median; [q_1_, q_3_]	18; [12, 24]
Absent (abs; %)	512; 73.6%	NA (abs; %)	21; 3.0%
Not typed (abs; %)	116; 16.7%	**Metastatic lymph-nodes**	
G1 (abs; %)	17; 2.4%	Median; [q_1_, q_3_]	0; [0, 2]
G2 (abs; %)	16; 2.3%	NA (abs; %)	14; 2.0%
G3 (abs; %)	24; 3.4%	**Surgery**	
NA (abs; %)	11; 1.6%	Mastectomy (abs; %)	268; 38.5%
**Multifocality**		Conserving-surgery (abs; %)	428; 61.5%
Absent (abs; %)	572; 82.2%	**Chemotherapy**	
Present (abs; %)	124; 17.8%	No (abs; %)	227; 32.6%
**Angioinvasion**		Yes (abs; %)	467; 67.1%
Absent (abs; %)	441; 63.4%	NA (abs; %)	2; 0.3%
Focal (abs; %)	130; 18.7%	**Chemotherapy scheme**	
Extensive (abs; %)	41; 5.9%	Not received (abs; %)	227; 32.6%
Not typed (abs; %)	84; 12.0%	C1 (abs; %)	119; 17.1%
**ER**		C2 (abs; %)	153; 22.0%
Median; [q_1_, q_3_]	60; [1, 90]	C3 (abs; %)	3; 0.4%
NA (abs; %)	8; 1.1%	C4 (abs; %)	112; 16.1%
**PgR**		C5 (abs; %)	76; 10.9%
Median; [q_1_, q_3_]	25; [0, 73]	NA (abs; %)	6; 0.9%
NA (abs; %)	9; 1.3%	**Chemotherapy completion**	
**ki67**		Yes (abs; %)	444; 63.8%
Median; [q_1_, q_3_]	20; [10, 39]	No (abs; %)	242; 34.8%
NA (abs; %)	16; 2.3%	NA (abs; %)	10; 1.4%
**Her2/neu**		**Trastuzumab**	
0 (abs; %)	193; 27.8%	No (abs; %)	600; 86.2%
1 (abs; %)	155; 22.3%	Yes (abs; %)	96; 13.8%
2 (abs; %)	90; 12.9%	**Trastuzumab completion**	
3 (abs; %)	99; 14.2%	Yes (abs; %)	84; 12.1%
NA (abs; %)	159; 22.8%	No (abs; %)	610; 87.6%
**Her2**		NA (abs; %)	2; 0.3%
Negative (abs; %)	445; 64.0%	**Hormone therapy**	
Positive (abs; %)	122; 17.5%	No (abs; %)	178; 25.6%
NA (abs; %)	129; 18.5%	Yes (abs; %)	512; 73.5%
**Grading**		NA (abs; %)	6; 0.9%
G1 (abs; %)	64; 9.2%	**Hormone therapy scheme**	
G2 (abs; %)	309; 44.4%	Not received (abs; %)	178; 25.6%
G3 (abs; %)	300; 43.1%	H1 (abs; %)	27; 3.9%
NA (abs; %)	23; 3.3%	H2 (abs; %)	4; 0.6%
**Diameter**		H3 (abs; %)	109; 15.7%
T1a (abs; %)	23; 3.3%	H4 (abs; %)	239; 34.3%
T1b (abs; %)	64; 9.2%	H5 (abs; %)	36; 5.2%
T1c (abs; %)	286; 41.1%	H6 (abs; %)	28; 4.0%
T2 (abs; %)	252; 36.2%	H7 (abs; %)	63; 9.0%
T3 (abs; %)	16; 2.3%	NA (abs; %)	12; 1.7%
T4 (abs; %)	31; 4.5%	**Hormone therapy completion**	
NA (abs; %)	24; 3.4%	Yes (abs; %)	387; 55.6%
**Lymph-node status**		No (abs; %)	286; 41.1%
N0 (abs; %)	361; 51.9%	NA (abs; %)	23; 3.3%

### An artificial intelligence-based approach for IDE prediction

We developed an artificial intelligence-based model to predict both 5-year and 10-year breast cancer IDE, after performing a stratified randomly sampling which allows to split both datasets in a hold-out training set, including 80% of patients, and a hold-out test set, comprising 20% of patients. The few missing data were initially estimated by means of a proximity algorithm which allows to replace the missing data of each patient with the data belonging to the patient having the minimum Euclidean distance from the patient under consideration and without missing values [33]. Potential missing features of newcome patients will be estimated comparing each incomplete observation to the 696 patients belonging to the dataset employed in this study.

**Fig 1:**
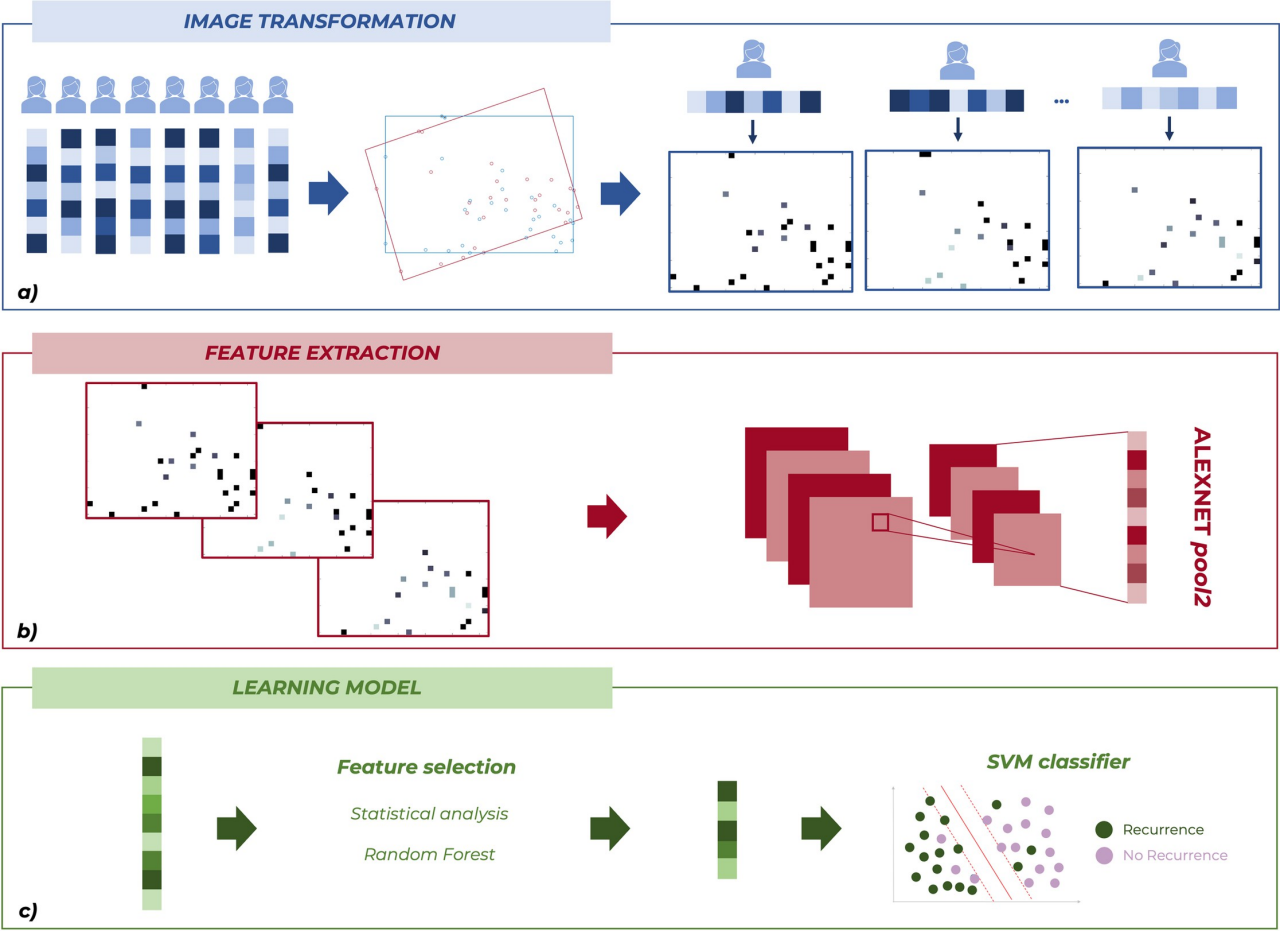
Schematic overview of the proposed approach. (a) After transforming each patient represented by a vector of clinical information to an image form, (b) from each image we extracted radiomic features by means of a pre-trained CNN. (c) Finally, we identified a subset of significative features, and we trained a SVM classifier to classify breast cancer patients in the two classes, namely, IDE and no IDE. All steps of the proposed approach have been implemented on both 5-year and 10-year datasets.

All steps of the proposed approach depicted in Fig 1 have been implemented on both 5-year and 10-year datasets. All the analysis steps have been performed by using MATLAB R2022a (Mathworks, Inc. Natick, MA, USA) software.

#### 1. Image transformation

The first step consisted in transforming each patient, represented by a vector of 28 clinical information, to an image form (Fig 1A). Given the hold-out training set as an array *X* of dimension *n* × *28*, where *n* represents the number of patients and *28* is the number of clinical features, we firstly computed X* as the transpose array of *X*. Then, we applied a *norm-1* normalization on X* with the purpose of normalizing each feature by its minimum and maximum. Further details about the normalization procedure are discussed in the work of Sharma et al. [34].

At this step, with the aim of defining a two-dimensional plane on which projecting pixel frames for each patient, we implemented a Kernel Principal Component Analysis (K-PCA) technique with gaussian kernel to reduce the feature space dimensionality [37]. In this way, we transformed a manifold in a *n*-dimensional space (one dimension for each patient) to a projection onto a two-dimensional plane, where each point represents the location of a single feature, where new feature coordinates are identified in the basis of the first two principal components. K-PCA rather than standard PCA has been implemented since decision boundaries of our data were described by non-linear functions.

Afterwards, the minimal bounding rectangle of all points representing feature locations in the plane was identified by means of a convex hull algorithm [38]. After receiving in input coordinates of all points, this algorithm allowed to define the rectangle having minimum area and including all points. This bounding rectangle along with all points representing feature locations were finally rotated in the horizontal direction.

The final step consisted in mapping feature values to feature locations, for each patient. To this end, we first transformed point coordinates in pixel frames to determine the location of every feature in the image-form. Then, for each patient, we created the corresponding image assigning to these locations the feature values for the patient into account. Accordingly, we generated a unique image for each patient. Further details about the pixel conversion procedure are discussed in the work of Sharma et al. [34].

Finally, on both hold-out test sets, this final step has been implemented starting from both the bounding rectangle and all feature locations previously identified by the related hold-out training set.

#### 2. Feature extraction

As depicted in Fig 1B, the second step consisted in extracting radiomic features from each image by means of a pre-trained Convolutional Neural Network (CNN), namely, AlexNET [39], after resizing all images to 227×227 pixels, since the network requires images of such size as input. Specifically, we extracted features from the pool2 layer of the network architecture which corresponds to the second pooling layer after the second convolutional layer of the network. The pool2 layer has an output with dimensions of 13×13×256 that is flattening to a single 43.264-length vector. As consequence, the number of extracted features is 43.264 in total for each image.

#### 3. Learning model

The final step consisted in developing a learning model able to predict both 5-year and 10-year breast cancer recurrence event (Fig 1C). Specifically, for each temporal frame, this model was originally evaluated on the hold-out training set within a 10-fold cross-validation scheme over 10 rounds, and afterward, the model was validated on the hold-out test set exploiting only features resulted as important within every cross-validation cycle of every round.

First, exploiting features collected in the previous step, we performed a feature selection procedure with the aim of identifying a subset of significative features. To start with, we separately investigated the predictive power of each feature by means of a nonparametric statistical test, retaining the only features whose p-value resulted less than 0.005. Then, we implemented a Random Forest-based feature selection process to overall evaluate the importance of features previously identified as statistically significant. Features were considered as important when their weight computed by means of the so-called Gini impurity was greater than the median weight of all features involved [40].

Subsequently, we classified breast cancer patients in the two classes, namely, recurrence and no recurrence, via a linear Support Vector Machine (SVM) classifier trained on the identified subset of significative features [41].

For each temporal frame, performances on both the hold-out training and the hold-out test set were evaluated in terms of Area Under the Curve (AUC) of the Receiver Operating Characteristic (ROC) curve and other standard metrics such as accuracy, sensitivity and specificity computed by identifying the optimal threshold by means of a Youden’s index test [42]. Finally, with the aim of determining the statistical significance of our results, for each of the two temporal frame we performed a permutation test to assess whether the classifier has found a real class structure in the data [43]. We calculated a null distribution of the AUC value of our model on dataset after randomly permuting the patient labels 100 times. Thus, we estimated an empirical p-value as the ratio of the number of times an AUC value computed with permuted labels was less than or equal to the actual AUC value increased by 1 over the number of implemented permutations increased by 1.

## Results

The stratified randomly sampling allowed to split both 5-year and 10-year datasets in a hold-out training set, including 80% of patients, and a hold-out test set, comprising 20% of patients. Consequently, the 5-year hold-out training set consisted of 557 patients, of which 136 (24.4%) IDE cases and 421 (75.6%) control cases, whereas the 5-year hold-out test consisted of 139 patients, of which 33 (23.7%) IDE cases and 106 (76.3%) control cases. Similarly, the 10-year hold-out training set counted 501 patients, of which 201 (40.1%) IDE cases and 300 (59.9%) control cases, and the 10-year hold-out test counted 125 patients, of which 50 (40.0%) IDE cases and 75 (60.0%) control cases.

**Fig 2:**
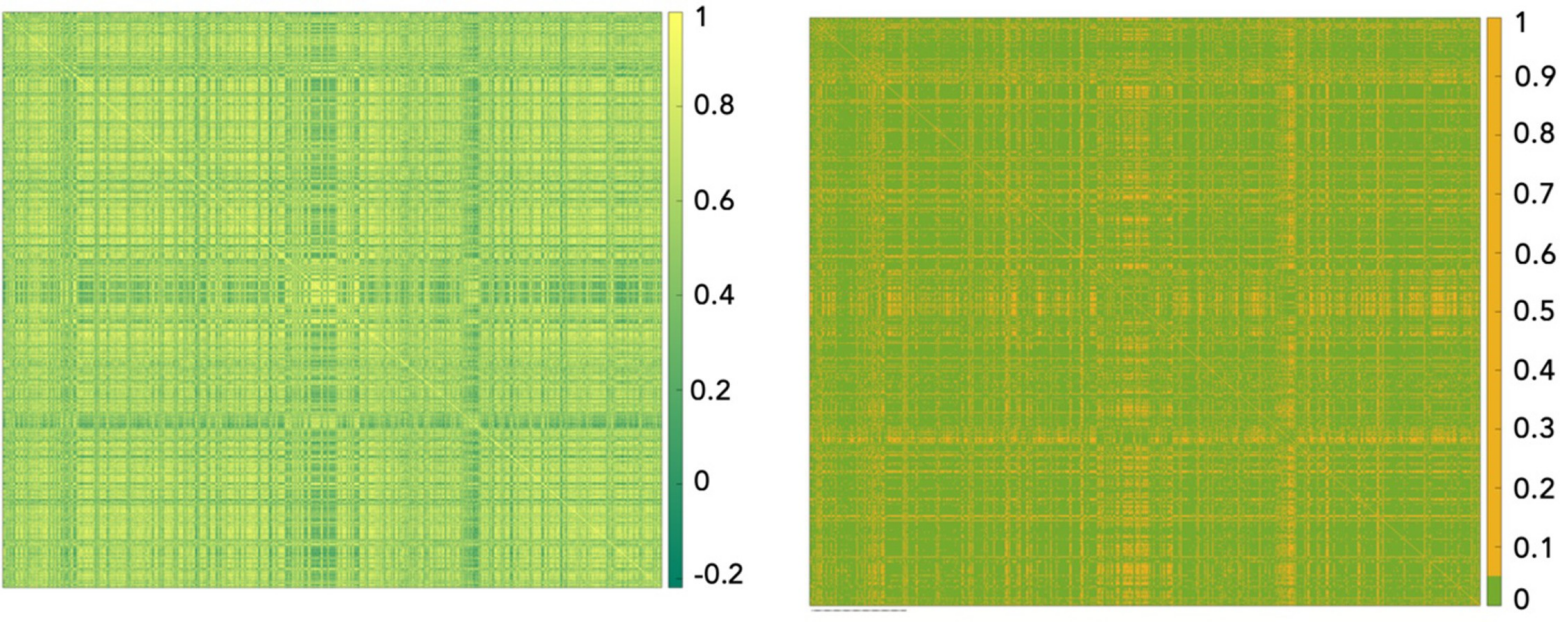
Correlation heatmap (a) computed through Spearman test and p-value heatmap (b) for patients belonging to the 5-year dataset. The correlation heatmap reported in Fig 2A demonstrates a strong positive correlation among patients, and the p-value heatmap depicted in Fig 2B confirms the statistical significance of these correlation.

To investigate existing relations among patients belonging to the same dataset, and, consequently, performing kernel PCA, we computed Spearman correlation coefficients among patients and the corresponding p-values [38]. As a result, two *n*×*n* matrices were obtained for each temporal frame. Fig 2 shows both correlation and p-value matrices associated to the 5-year dataset. The correlation heatmap show a significatively strong positive correlation among patients. Results related to the 10-year dataset are approximately the same and, therefore, are shown in a S1 Fig to not burden the discussion.

**Fig 3:**
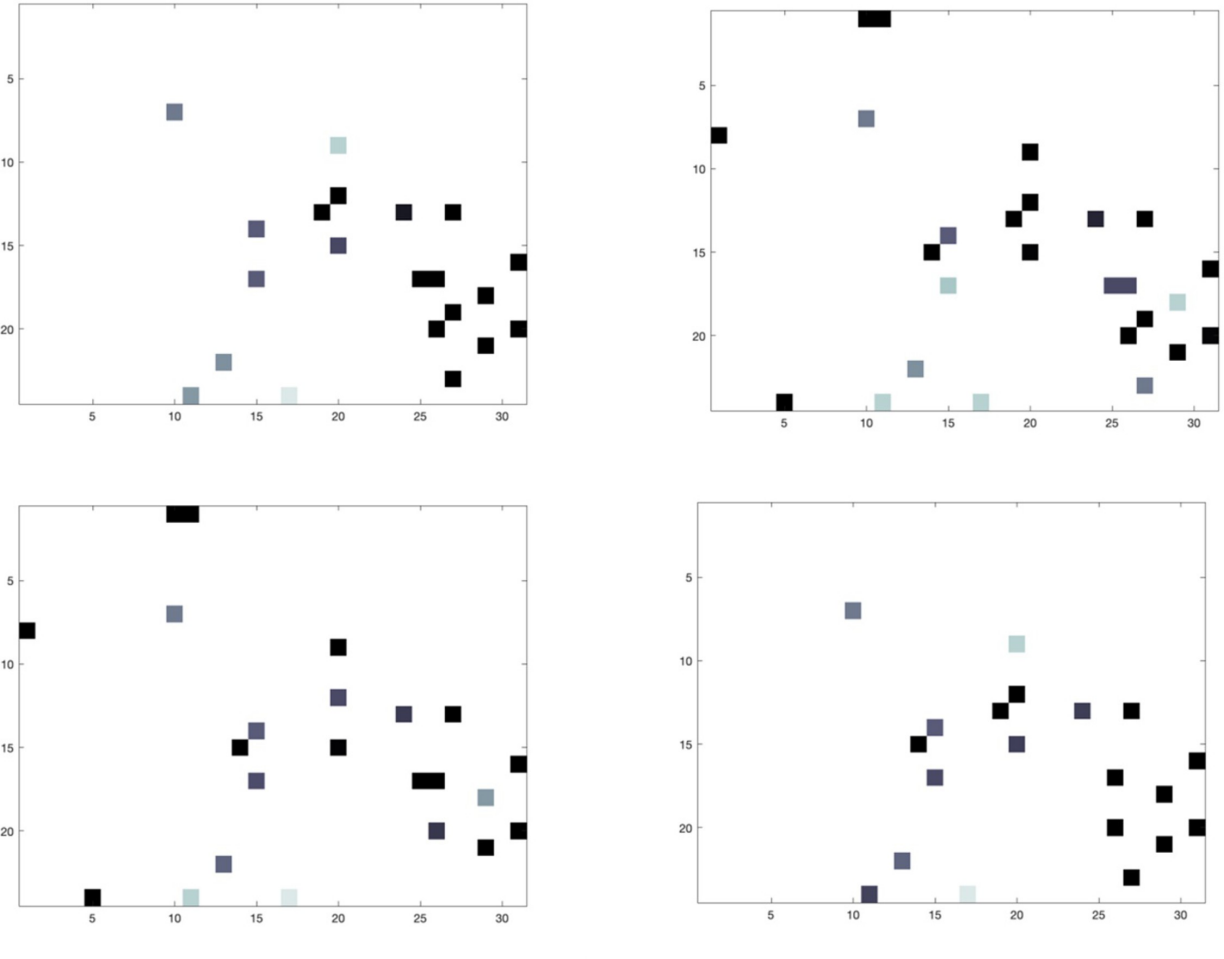
Examples of images generated within the image transformation procedure for patients belonging to the 5-year dataset. Images (a) and (b) referred to non-IDE patients, whereas images (c) and (d) belonged to IDE patients.

Then, the image transformation procedure allowed to generate a unique image for each patient, mapping feature values of every patient to feature locations previously identified. Fig 3 provides some examples of generated images.

Images provided by Fig 3 highlight both similarities between patients belonging to the same class, and discrepancies between patients of different classes, in terms of pixel intensity values. Accordingly, examples of images computed for patients included in the 10-year dataset emphasize the same similarities and discrepancies and are depicted in a S2 Fig to not burden the discussion.

**Fig 4:**
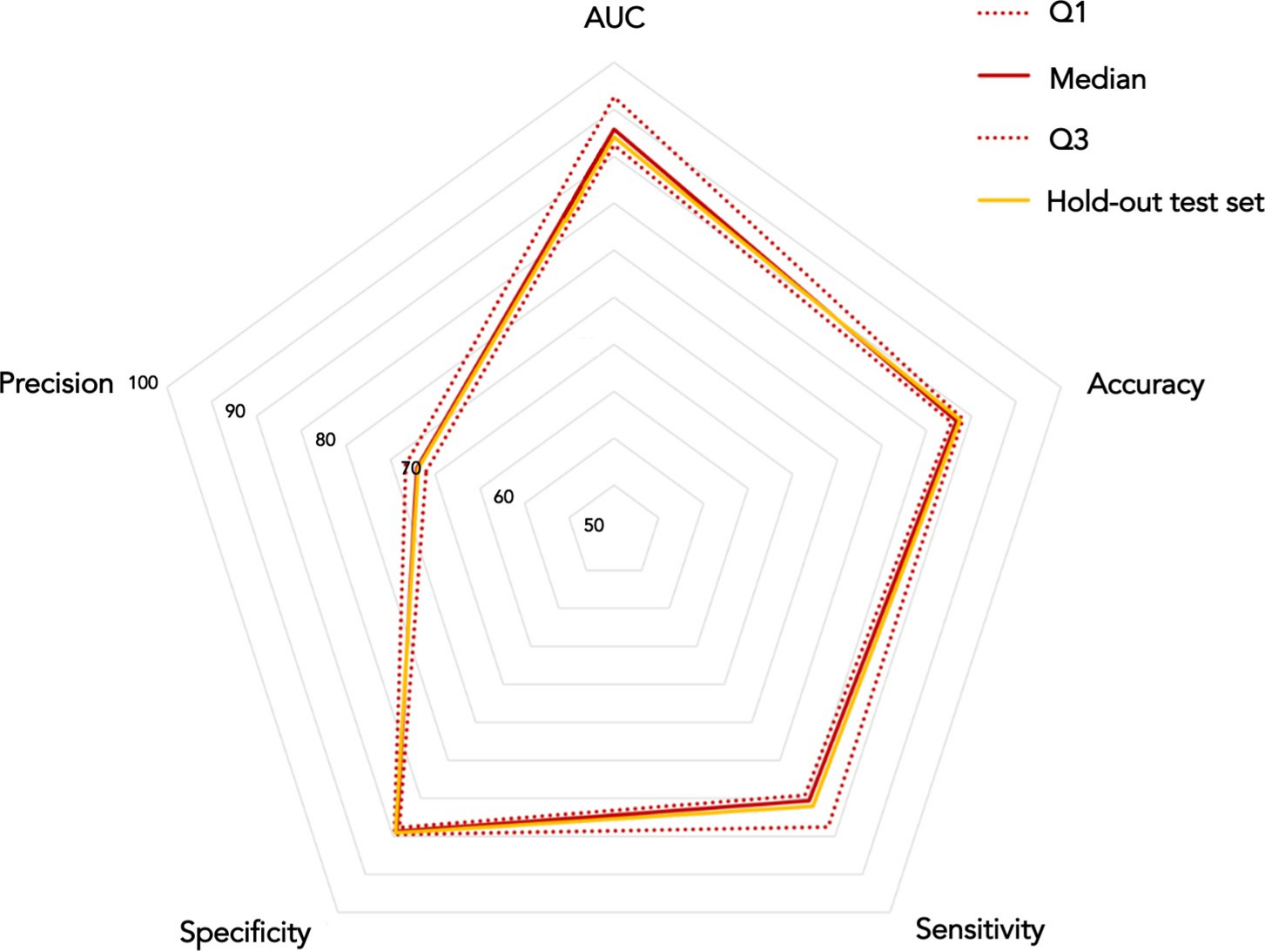
Radar chart depicting classification performances achieved by the 5-year predictive model on both hold-out training and hold-out test set. On the hold-out training set, performances were evaluated in terms of percentage median values and the first and the third quartile values. Reported percentages refers to results achieved on the hold-out test set.

**Fig 5:**
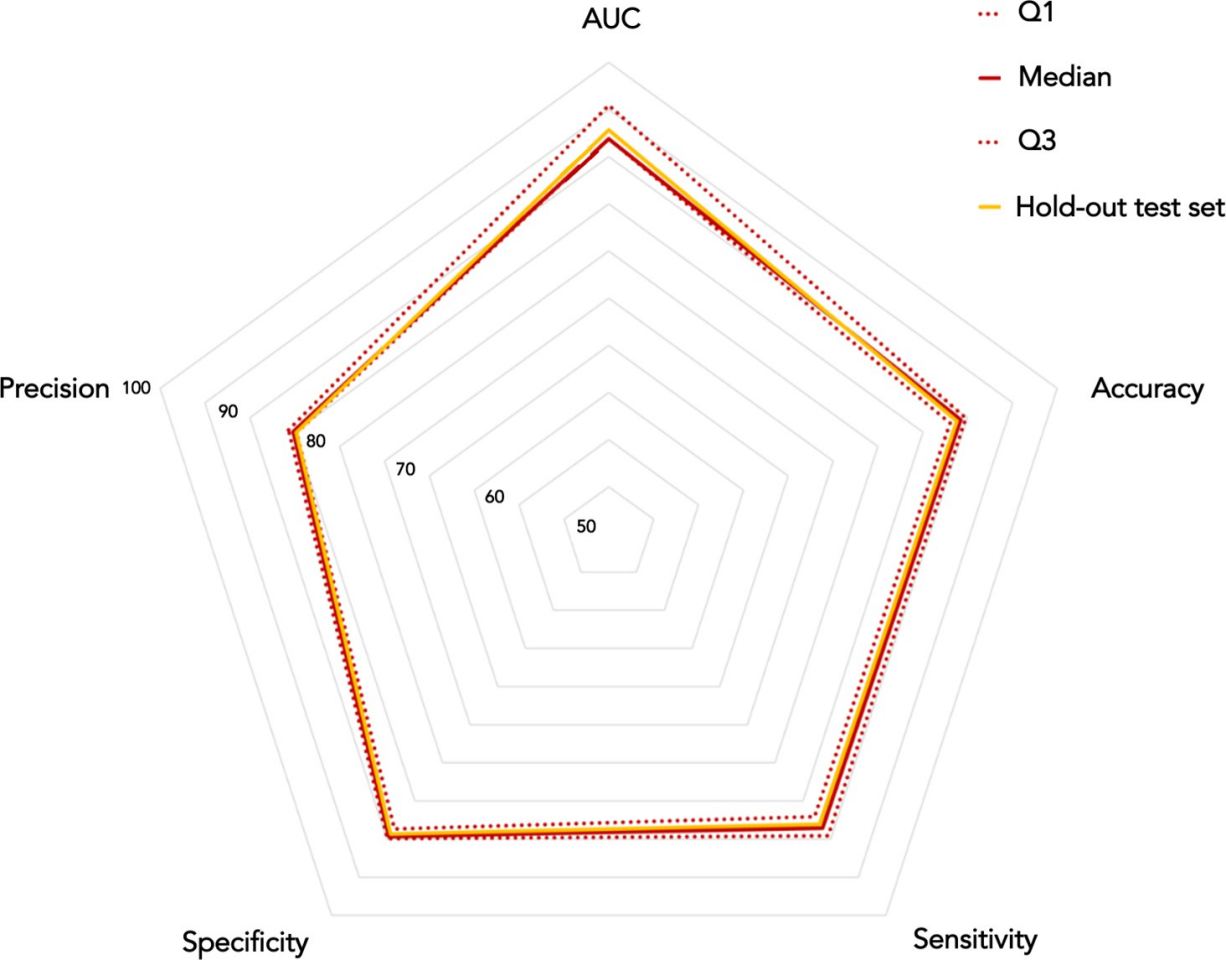
Radar chart depicting classification performances achieved by the 10-year predictive model on both hold-out training and hold-out test set. On the hold-out training set, performances were evaluated in terms of percentage median values and the first and the third quartile values. Reported percentages refers to results achieved on the hold-out test set.

Classification performances achieved by both 5-year and 10-year predictive model are presented in Figs 4 and 5, respectively. Specifically, each radar chart includes performances obtained on both the hold-out training and the hold-out test set.

Both 5-year and 10-year models resulted particularly accurate in predicting breast cancer recurrence event, achieving on the hold-out test set a percentage accuracy equals to 88.71% and 88.82%, respectively. Besides, these performances reveal a perfect balance between the number of IDE cases correctly classified as IDE, i.e., the sensitivity percentage value, and the number of non-IDE cases correctly classified as non-IDE, i.e., the specificity percentage value. Radar charts also revealed classification performances achieved on the hold-out test set fall in the confidence interval calculated on performances reached on the hold-out training set.

The statistical significance of these results was confirmed by the outcome of the permutation test. Specifically, the permutation test confirmed that the AUC value predicted on the independent test does not belong to the null distribution of the AUC values obtained after randomly permuting the patient labels 100 times (p-value equal to 1 for each of two temporal frame). Therefore, the performed models have found a real class structure in the data.

## Discussion

Breast cancer prognosis has always been adversely affected by the molecular heterogeneity of this tumor, precluding the design of targeted treatments for each tumor type [3, 4, 44, 45]. So far, several machine learning algorithms have been developed by the scientific community to support both the diagnosis of breast cancer and the clinical decision-making process in breast cancer treatment [14, 15]. Though these models achieved encouraging results also in predicting the occurrence of an IDE after a primary breast tumor, such as recurrence, metastasis, contralateral and second tumors, performances were not suitable for clinical practice [8, 46].

As documented by recent reviewers on the topic, the models proposed in literature to predict disease recurrence that use only clinical data are very few (Table 2). Recently, in the work of Fu et al. [46], authors developed a 5-year machine learning survival model exploiting demographics, pathological, diagnostics and therapeutic information of patients in their primary structured form. We also proposed different machine learning models to predict a breast cancer IDE analyzing the same dataset, though fewer in number due to the subsequent sample repopulation. We first developed an advanced ensemble algorithm able to identify the so-called confounding patients, having the same clinical and histological characteristics as other patients but responding differently to the adjuvant treatment [33]. Afterwards, we implemented an explainable artificial intelligence framework which offers clinicians a useful white tool for the interpretation of the predicted classification results. This algorithm was then optimized developing a complex grid search algorithm [30]. However, these models were not yet suitable to be implement in clinical practice. On ten independent tests, we achieved a median AUC of 77.1% and 76.3% and a median accuracy value of 75.5% and 71.3% for the 5-year follow-up and the 10-year follow-up, respectively.

**Table 2:** Performance of the state-of-the-art models aimed at predicting a breast cancer invasive disease event by means of clinical features alone.

	Performances (AUC, %)
Fu et al. (2019) [46]	10-years: 84.51%
Massafra et al. (2022) [30]	5-years: 77.10%
	10-years: 76.30%
Massafra et al. (2023) [33]	5-years: 75.00%
	10-years: 70.00%
Our best proposed model	5-years: 92.07%
	10-years: 92.84%

With the aim of overcoming limits of our previous efforts in predicting the occurrence of a second breast cancer IDE by means of machine learning techniques, in this work, we proposed a novel approach exploiting the well-known capabilities of pre-trained CNNs in analyzing images to extract low-level quantitative imaging features from images obtained transforming each patient, represented by a vector of clinical information, to an image form. Thus, we developed a transfer-learning approach to predict the occurrence of an IDE at both 5-year and 10-year follow-ups. For this purpose, we considered a private database comprising 696 female patients with a first invasive breast cancer diagnosis and whose clinical and histological information was known. Then, we transformed the feature vector of each patient to an image form, and we analyzed them by means of a pre-trained CNN. Finally, we classified breast cancer patients in the two classes, namely, IDE and non-IDE, via a SVM classifier trained on a subset of significative features previously identified. Performance achieved by both 5-year and 10-year models resulted particularly accurate in predicting breast cancer recurrence event, also revealing a perfect balance between the number of IDE cases correctly classified as IDE, and the number of non-IDE cases correctly classified as non-IDE. Indeed, the 5-year and 10-year predictive models achieved an AUC value of 92.07% and 92.84%, an accuracy of 88.71% and 88.82%, a sensitivity of 86.83% and 88.06%, a specificity of 89.55% and 89.3% respectively.

The proposed approach allowed to significantly improve classification performances, despite the presence of confounding patients. The absence of patients with a strong negative correlation, thus presenting relevant differences in terms of clinical and histological characteristics, was highlighted by the correlation heatmaps (Figs 2A and S1A) and was then confirmed as statistically significant by the p-value heatmaps (Figs 2B and S1B). Hence, our study overcome biases resulting from the presence of confounding patients, being the first one proposing an approach which converts clinical data commonly collected in clinical practice into an image-form, by computing similarity measures among patients and, thus, detecting information not captured by common machine learning-based models. Moreover, this is the first time this approach is employed for predicting an IDE after a breast cancer diagnosis.

The proposed tool developed certainly has the advantage of being user-friendly and low-cost, as it only uses clinical characteristics commonly and directly collected in clinical practice by the medical oncologist in charge of the patient. Other methods proposed at the state-of-the-art, however, achieve performances comparable to those obtained by the proposed model but using other types of feature [22], such as radiomics and genomics, which may not be readily available to the medical oncologist for the assessment of the risk of recurrence.

However, this work has the limitation of losing in explainability with respect to our previous studies. Therefore, our future works will include both a validation process of the proposed model, and the implementation of an explainable artificial intelligence framework which can provide a more transparent assessment of the classification result, driving clinicians to be more confident in enclosing these artificial intelligence-based tools in clinical practice.

## Conclusions

Despite the heterogeneous nature of our experimental population caused by the unavoidable evolution of oncological treatments, as well as of diagnostic procedures, our approach represents a step towards the definition of a deep learning-based decision support system for identifying patients at high risk of occurrence of an invasive disease event, exploiting CNNs to analyze clinical and histopathological information commonly collected in clinical practice and converted to an image form. The proposed model provides high-performing results with respect to state-of-the-art models which exploit raw data to train common machine learning algorithms. Thus, this method would allow clinicians to discriminate between low-risk and high-risk patients in a non-invasive and inexpensive way, with the aim of defining personalized oncological therapeutic treatments.

## Supporting information

S1 Fig Correlation matrix heatmap (a) computed through Spearman test and p-value matrix heatmap (b) for patients belonging to the 10-year dataset.
(PDF)

S2 Fig Examples of images generated within the image transformation procedure for patients belonging to the 10-year dataset. Images (a) and (b) referred to non-IDE patients, whereas images (c) and (d) belonged to IDE patients.
(PDF)
