# Supplementary material for: Prediction of breast cancer Invasive Disease Events using transfer learning on clinical data as image-form
Source: PLoS One. 2024 Nov 21;19(11):e0312036. doi: 10.1371/journal.pone.0312036 (PMC11581389; doi:10.1371/journal.pone.0312036)
Supplement: S1 Fig — Correlation matrix heatmap (a) computed through Spearman test and p-value matrix heatmap (b) for patients belonging to the 10-year dataset. (PDF) [file pone.0312036.s001.pdf]

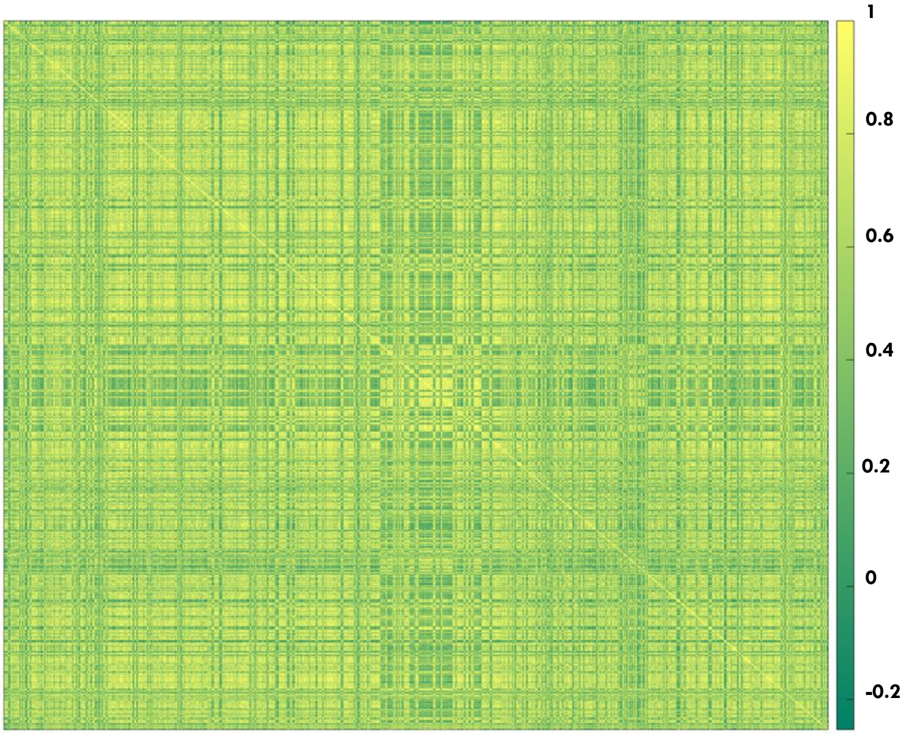

(a)

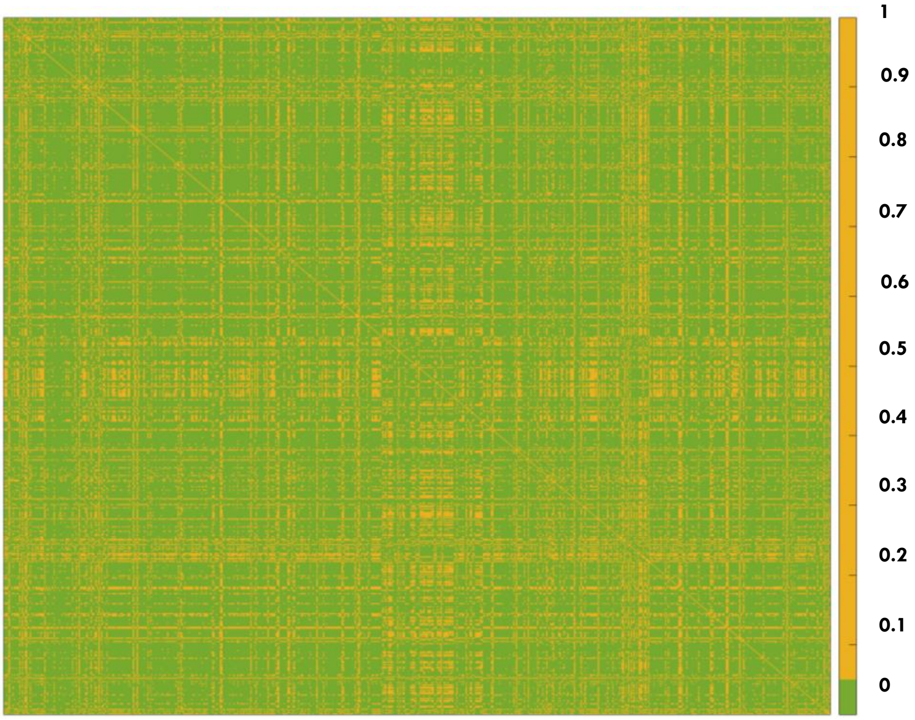

(b)

Figure S1. Correlation matrix heatmap (a) computed through Spearman test and p-value matrix heatmap (b) for patients belonging to the 10-year dataset.
