# Supplementary material for: Prediction of breast cancer Invasive Disease Events using transfer learning on clinical data as image-form
Source: PLoS One. 2024 Nov 21;19(11):e0312036. doi: 10.1371/journal.pone.0312036 (PMC11581389; doi:10.1371/journal.pone.0312036)
Supplement: S2 Fig — Images (a) and (b) referred to non-IDE patients, whereas images (c) and (d) belonged to IDE patients. (PDF) [file pone.0312036.s002.pdf]

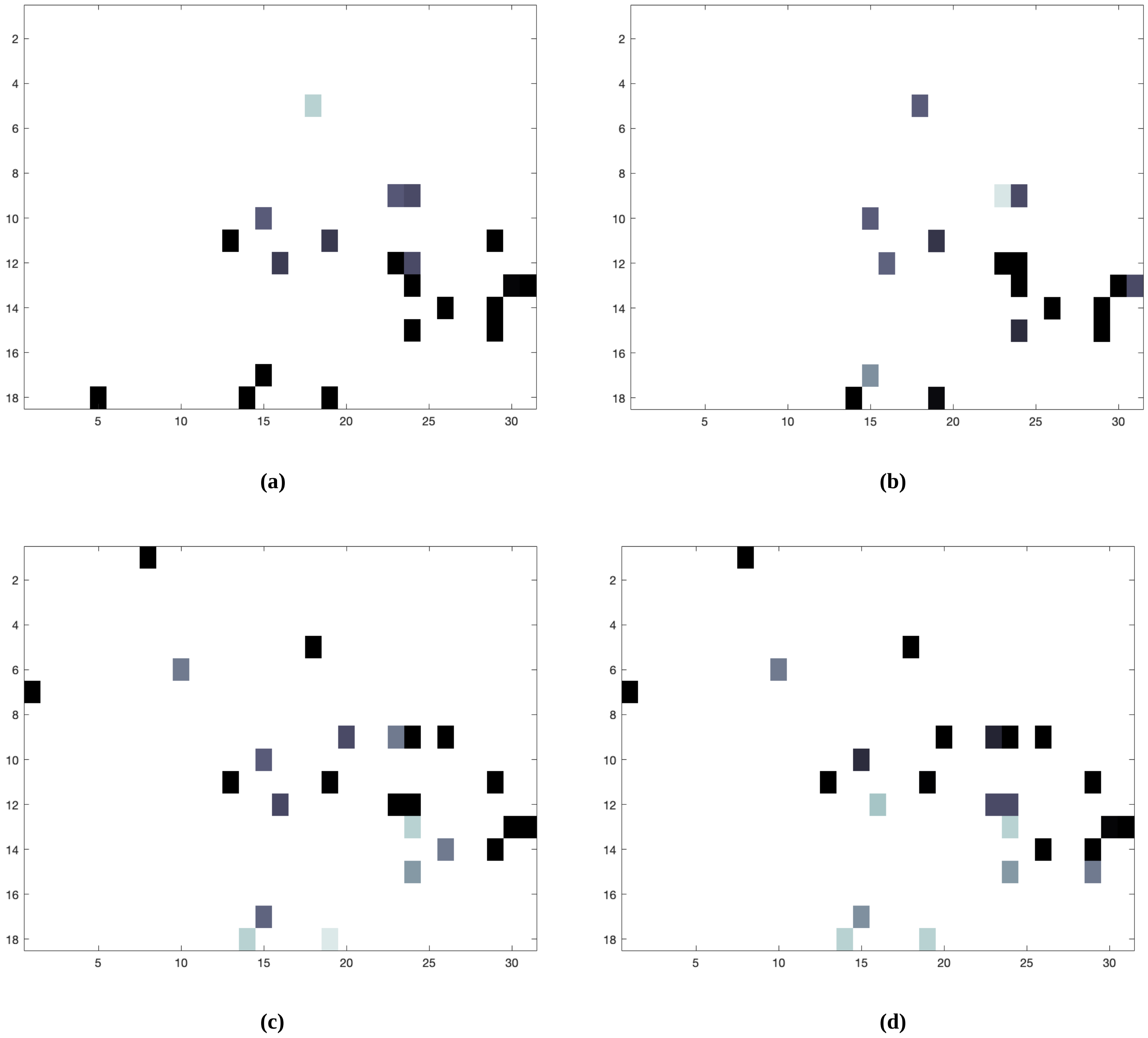

Figure S2. Examples of images generated within the image transformation procedure for patients belonging to the 10-year dataset. Images (a) and (b) referred to non-IDE patients, whereas images (c) and (d) belonged to IDE patients.
